# Supplementary material for: Genetically encoded tool for manipulation of ATP/ADP ratio in human cells
Source: bioRxiv. 2025 Aug 23:2025.08.12.670003. Preprint. [Version 2] doi: 10.1101/2025.08.12.670003 (PMC12393285; doi:10.1101/2025.08.12.670003)
Supplement: Supplement 5 [file NIHPP2025.08.12.670003v2-supplement-5.pdf]

bioRxiv preprint doi: https://doi.org/10.1101/2025.08.12.670003; this version posted August 23, 2025. The copyright holder for this preprint (which was not certified by peer review) is the author/funder, who has granted bioRxiv a license to display the preprint in perpetuity. It is made available under aCC-BY 4.0 International license.

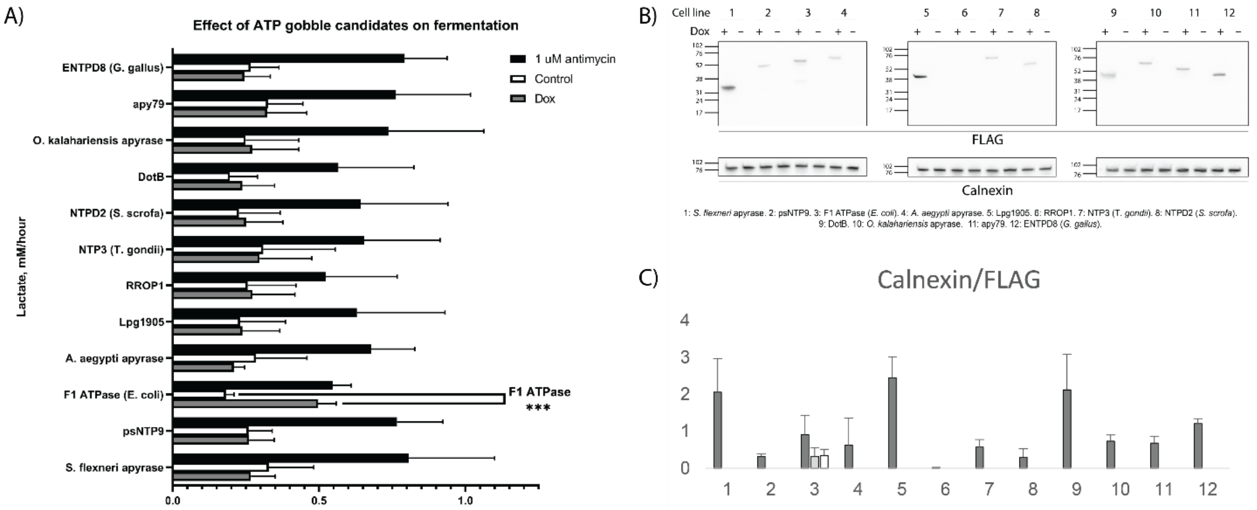

Figure S1. Screening for ATPGobble candidates. 1: RSFA (*S. flexneri* apyrase). 2: psNTP9. 3: F1-ATPase. 4: *A. aegypti* apyrase. 5: Lpg1905. 6: RROP1. 7: NTP3. 8: NTPD2. 9: DotB. 10: *O. kalahariensis* apyrase. 11: apy79. 12: NTPD8. A) Lactate assay showing no ATPGobble activity for most candidates except F1 ATPase from E. coli. B) Western blot showing the expression levels of the FLAG-tagged candidates used for the lactate assay and C) Western blot quantification (n = 3). Experiment means + standard deviation of experiment means.

bioRxiv preprint doi: https://doi.org/10.1101/2025.08.12.670003; this version posted August 23, 2025. The copyright holder for this preprint (which was not certified by peer review) is the author/funder, who has granted bioRxiv a license to display the preprint in perpetuity. It is made available under aCC-BY 4.0 International license.

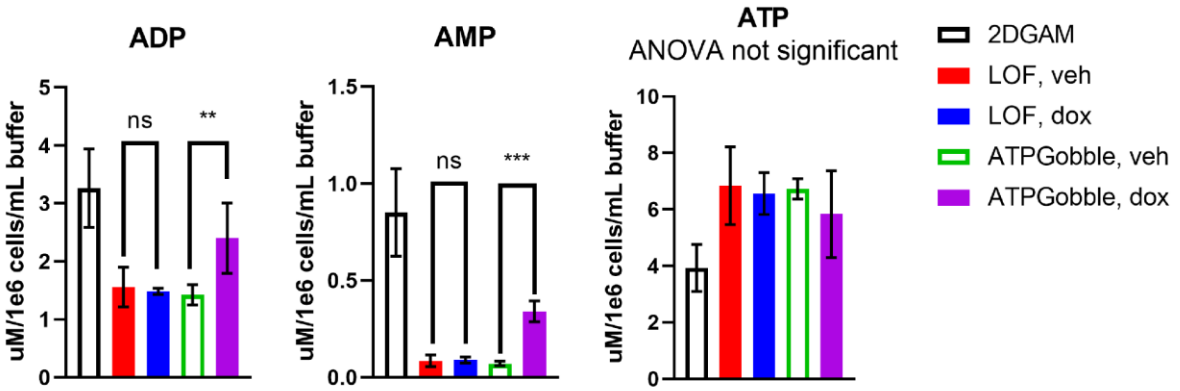

Figure S2. ATPGobble expression in hTERT-RPE1 increases the concentrations of ADP and AMP, while ATP is maintained at a stable level. 3 LC/MS experiments 5 biological replicates each. Repeated measurements ANOVA followed by the main row effect test with Tukey's adjustment for multiple comparisons. ns > 0.05, * < 0.05, ** < 0.01 *** < 0.001. dox: doxycycline, veh: vehicle. Experiment means ± standard deviation of experiment means.

bioRxiv preprint doi: https://doi.org/10.1101/2025.08.12.670003; this version posted August 23, 2025. The copyright holder for this preprint (which was not certified by peer review) is the author/funder, who has granted bioRxiv a license to display the preprint in perpetuity. It is made available under aCC-BY 4.0 International license.

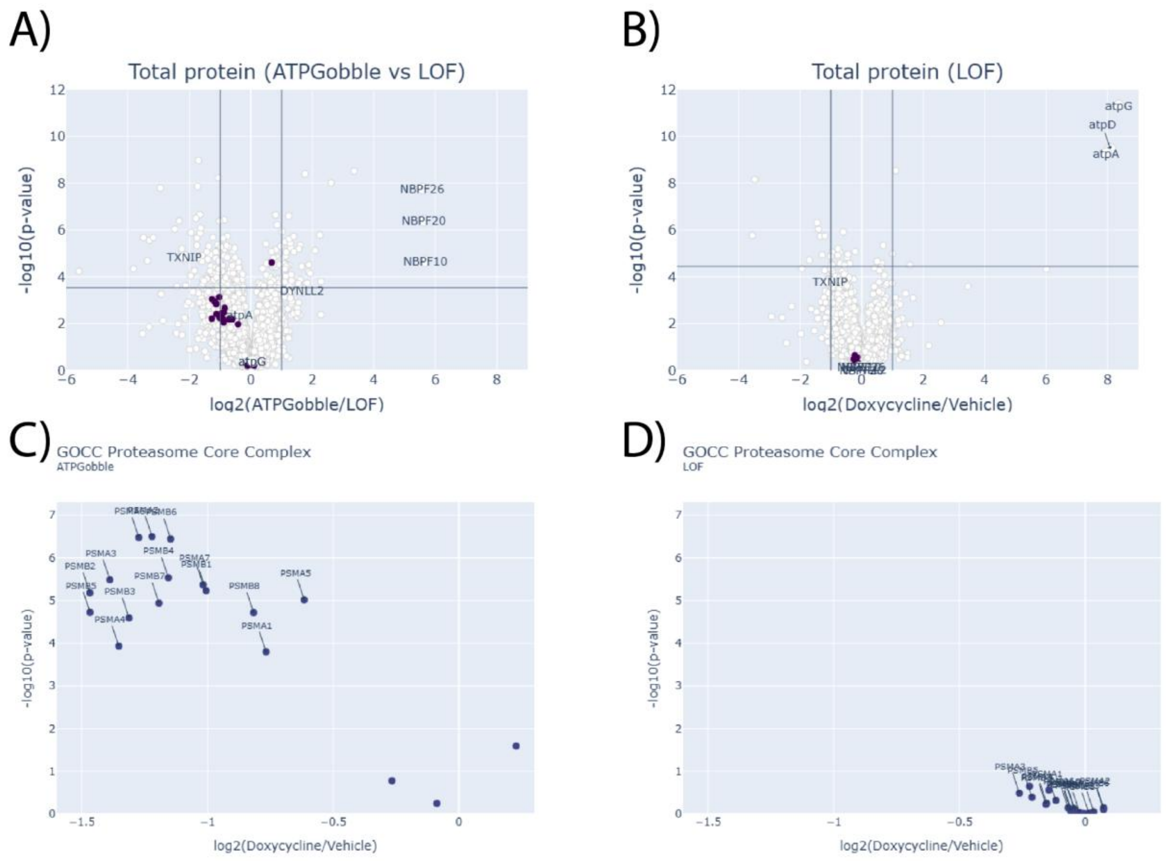

Figure S3. Alternative comparisons of total proteomics data and breakdown of proteasome core data set. A) Volcano plot comparing doxycycline treated hTERT-RPE1-LOF and ATPGobble cells. B) Volcano plot comparing vehicle vs doxycycline treated hTERT-RPE1-LOF cells. Volcano plots showing the changes in the expression of proteasome core complex proteins after vehicle vs doxycycline treatment in hTERT-RPE1-ATPGobble (C) vs LOF (D) cells.

bioRxiv preprint doi: https://doi.org/10.1101/2025.08.12.670003; this version posted August 23, 2025. The copyright holder for this preprint (which was not certified by peer review) is the author/funder, who has granted bioRxiv a license to display the preprint in perpetuity. It is made available under aCC-BY 4.0 International license.

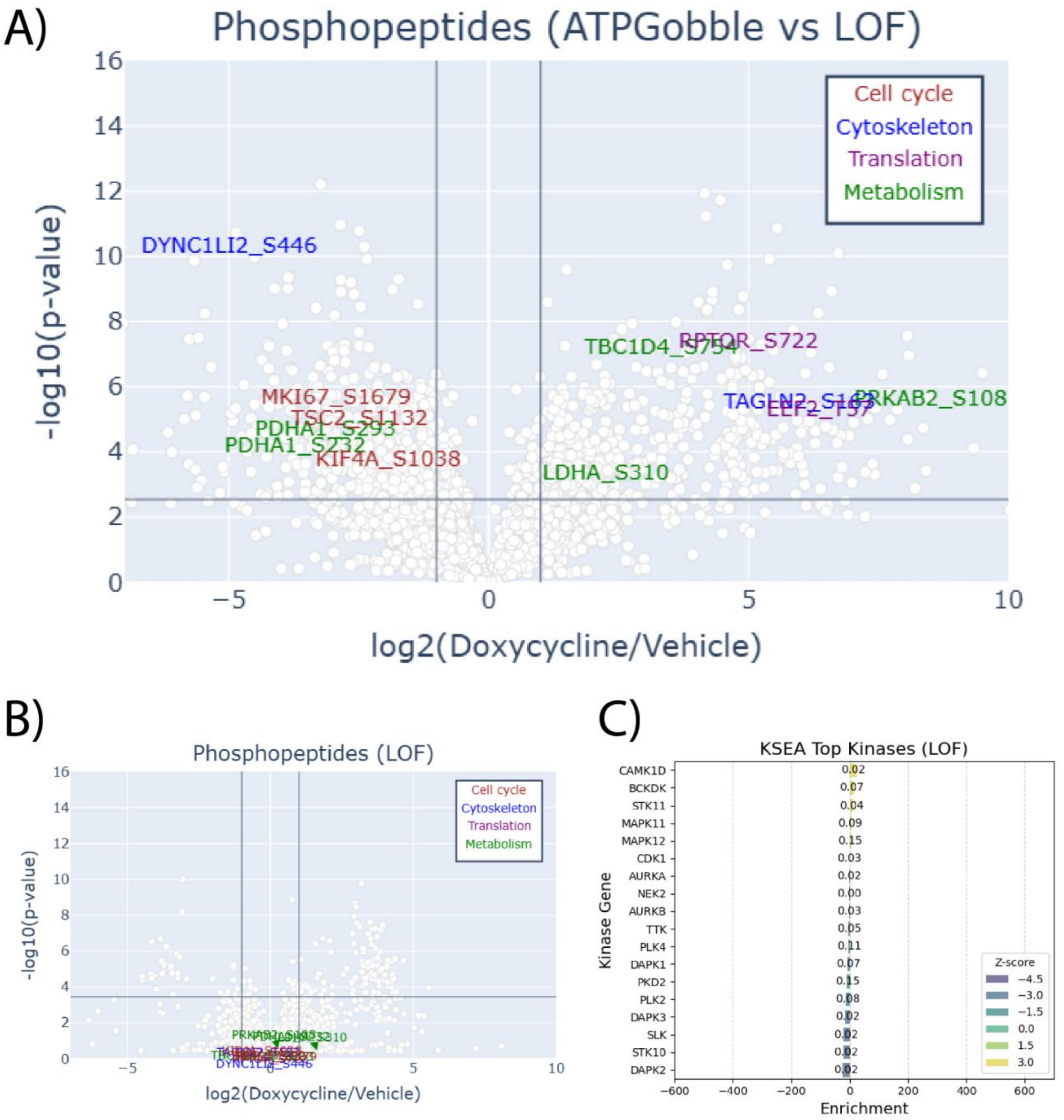

Figure S4. ATPGobble activity results in phosphoproteomic changes – supplementary analyses. A) A volcano plot comparing phosphopeptides from hTERT-RPE1-LOF and hTERT-RPE-ATPGobble cells after doxycycline treatment. B) A volcano plot comparing hTERT-RPE1-LOF cells treated with vehicle vs doxycycline. The targets and the axis ranges shown in A and B are the same as in Figure 5A. C) KSEA of hTERT-RPE1-LOF cells after vehicle vs doxycycline treatment. The X axis range is the same as in Figure 5C.

bioRxiv preprint doi: https://doi.org/10.1101/2025.08.12.670003; this version posted August 23, 2025. The copyright holder for this preprint (which was not certified by peer review) is the author/funder, who has granted bioRxiv a license to display the preprint in perpetuity. It is made available under aCC-BY 4.0 International license.

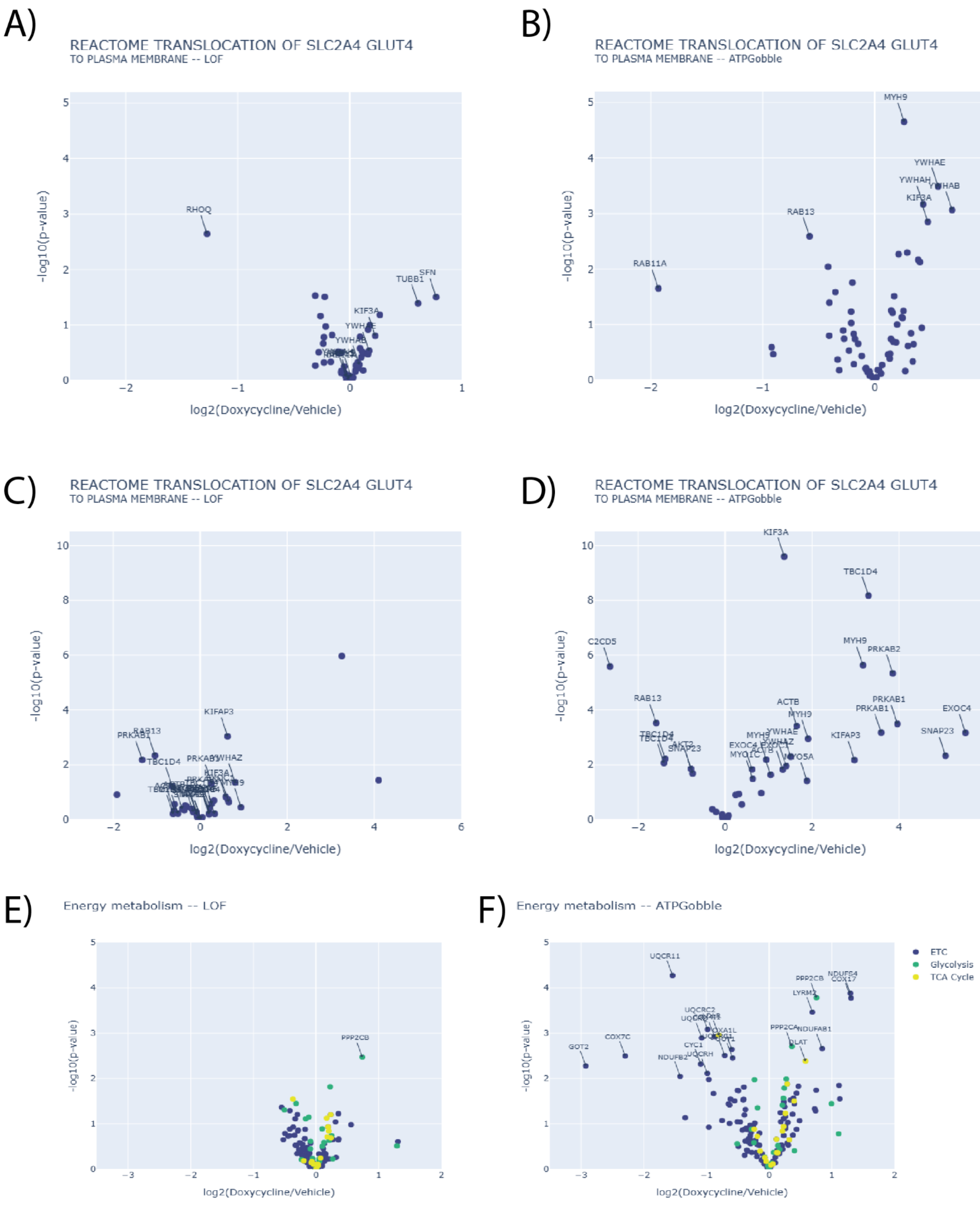

Figure S5. Changes in energy-producing activity in ATPGobble-expressing cells. Total-protein changes in proteins regulating the translocation of GLUT4 (coded by *SLC2A4*) to the membrane in hTERT-RPE1-LOF (A) and hTERT-RPE1-ATPGobble (B) cells after vehicle vs doxycycline treatment. Phosphopeptide changes of the same gene set in hTERT-RPE1-LOF

bioRxiv preprint doi: https://doi.org/10.1101/2025.08.12.670003; this version posted August 23, 2025. The copyright holder for this preprint (which was not certified by peer review) is the author/funder, who has granted bioRxiv a license to display the preprint in perpetuity. It is made available under aCC-BY 4.0 International license.

(C) and hTERT-RPE1-ATPGobble (D) cells after vehicle vs doxycycline treatment. Changes in genes coding for ETC, glycolysis and TCA cycle machinery, LOF (E) and ATPGobble (F).

bioRxiv preprint doi: https://doi.org/10.1101/2025.08.12.670003; this version posted August 23, 2025. The copyright holder for this preprint (which was not certified by peer review) is the author/funder, who has granted bioRxiv a license to display the preprint in perpetuity. It is made available under aCC-BY 4.0 International license.

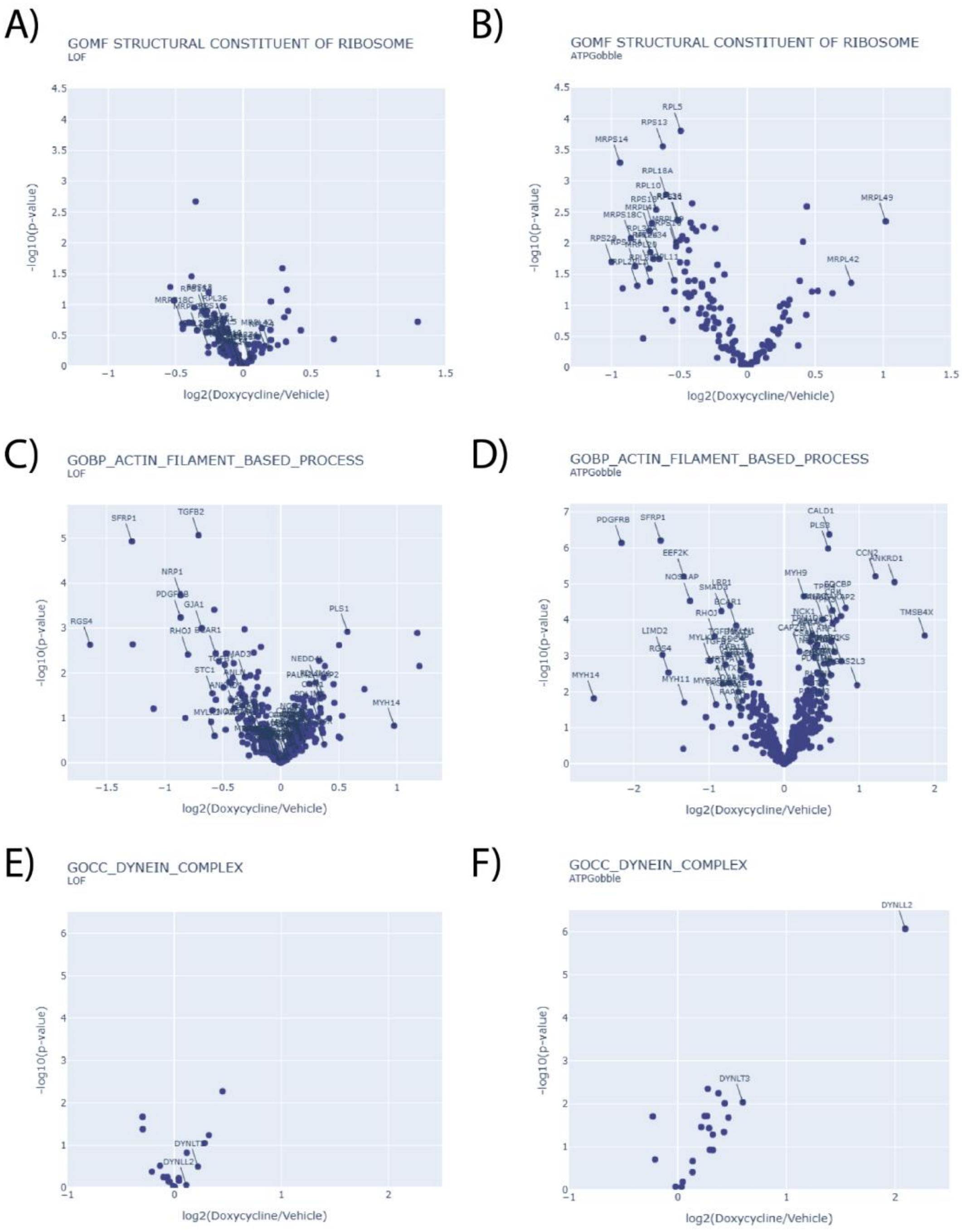

Figure S6. Changes in energy-consuming activity in cells expressing ATPGobble. Changes in GOMF STRUCTURAL CONSTITUENT OF RIBOSOME proteins in hTERT-RPE1-LOF (A) and hTERT-RPE1-ATPGobble (B) cells after vehicle vs doxycycline treatment. Changes in GOBP ACTIN FILAMENT BASED PROCESS proteins in hTERT-RPE1-LOF (C) and hTERT-RPE1-ATPGobble (D) cells after vehicle vs doxycycline treatment. Changes in GOCC DYNEIN COMPLEX proteins in hTERT-RPE1-LOF (E) and hTERT-RPE1-ATPGobble (F) cells after vehicle vs doxycycline treatment.

bioRxiv preprint doi: https://doi.org/10.1101/2025.08.12.670003; this version posted August 23, 2025. The copyright holder for this preprint (which was not certified by peer review) is the author/funder, who has granted bioRxiv a license to display the preprint in perpetuity. It is made available under aCC-BY 4.0 International license.

Table S1. Cell line generation and seeding density for cell growth comparison

Cell line	Zeocin, ug/mL	Blasticidin, ug/mL	Puromycin, ug/mL	Cells/well for 96-well plate growth assay
hTERT-RPE1	100	5	20	400
MRC5	150	5	1	1000
IMR90	40	10	2	1000
A549	100	5	2	400
HeLa	100	5	1	1000
HCT116	100	8	0.8	200

bioRxiv preprint doi: https://doi.org/10.1101/2025.08.12.670003; this version posted August 23, 2025. The copyright holder for this preprint (which was not certified by peer review) is the author/funder, who has granted bioRxiv a license to display the preprint in perpetuity. It is made available under aCC-BY 4.0 International license.

Table S2. Summary of steps for the tail-end imputation.

Data set	Condition	Impute with
Total proteomics (report.pg_matrix.tsv)	All values in dox/veh group and no values in the opposite group.	minimum*0.2*(1-abs(random.gauss(0, 0.5))) (try again if the generated number below 0)
Total proteomics (report.pg_matrix.tsv)	4 or more missing values in dox/veh group and at most 1 missing value in the opposing group.	minimum*(1-abs(random.gauss(0, 0.25))) (try again if the generated number below 0)
Phosphopeptides (report.phosphosites_99.tsv)	At least 3 non-NaN values in dox/veh group and all NaN values in the opposing group	minimum*0.2*(1-abs(random.gauss(0, 0.5))) (try again if the generated number below 0)
Phosphopeptides (report.phosphosites_99.tsv)	At least 4 non-NaN values in dox/veh group and less than 3 non-NaN values in the opposing group.	minimum*(1-abs(random.gauss(0, 0.25))) (try again if the generated number below 0)
